# Perceptions of Community Risk and Travel During Pregnancy in an Area of Zika Transmission

**DOI:** 10.7759/cureus.1516

**Published:** 2017-07-26

**Authors:** Neeraja Chandrasekaran, Mabel Marotta, Sabrina Taldone, Christine Curry

**Affiliations:** 1 Public Health Sciences, University of Miami Miller School of Medicine; 2 Department of Obstetrics and Gynecology, University of Miami Miller School of Medicine/Jackson Memorial Hospital, Florida, USA; 3 Department of Internal Medicine, University of Miami Miller School of Medicine/Jackson Memorial Hospital, Florida, USA

**Keywords:** zika virus, pregnancy, public health, patient education, zika, prevention

## Abstract

Introduction

Between June 2016 and August 2016, the first cases of the Zika transmission were reported in Miami, FL, US. Since then, travel advisories have been issued by the Centers for Disease Control and Prevention (CDC) to avoid travel to Florida. Women that are of childbearing age or pregnant are the populations most vulnerable to Zika infection because birth defects can occur in infants born to infected mothers. Till date, there are no studies assessing the perception of the community risk of Zika in pregnant women residing in an affected region such as Miami.

Methods

A cross-sectional design was utilized for this study. The survey included questions assessing community risk and travel perceptions. Surveys were distributed in the antenatal clinics at the University of Miami Hospital and the Jackson Memorial Hospital.

Results

A total of 85 women were surveyed between January 27, 2016 and March 3, 2017. Of the surveyed women, 92.6 percent believed Zika is an important issue in their community, 85.9 percent reported a change in behavior because of Zika, 26.9 percent believed they can get Zika at their location, and 13.9 percent considered moving away from Florida because of Zika.

Conclusion

Despite the majority of women believing Zika is an important issue in their community, only one-fourth believed they could get Zika in Miami. Efforts to educate pregnant women in affected areas about preventive measures against the Zika infection should be undertaken. Further studies comparing the perceptions of community risks and travel behaviors in other affected areas are warranted.

## Introduction

The Zika virus is a member of the Flaviviridae family that uses mosquito vectors for disease transmission [1]. It poses a risk to women of childbearing age and pregnant women because it can be transmitted vertically via the transplacental route or during childbirth [1]. Infants born to infected mothers have been reported to suffer from numerous birth defects, including microcephaly, intracranial and cerebral malformations, ocular dysfunction, brainstem dysfunction, difficulties in swallowing, and fetal demise [1-2]. 

Between June 2016 and August 2016, the first cases of Zika transmissions occurred in the US in Miami, FL [3]. Since then, the Centers for Disease Control and Prevention (CDC) issued recommendations for possible infection with Zika during pregnancy and also issued advisories to avoid traveling to Miami from August 1, 2017 to June 2, 2017 [4]. Studies assessing knowledge, attitudes, practices, and travel risks have been published in unaffected areas [5-6]. Till date, there are no studies assessing the perceptions of community risk and travel behaviors in pregnant women residing in affecting areas of the US, despite this population being the most vulnerable to infection. In this study, we aim to delineate the differences in the perception of community risk during pregnancy in an affected area.

## Materials and methods

A cross-sectional design was utilized to perform this study. Following Institutional Review Board approval, a survey was distributed to the antenatal clinics of the Jackson Memorial Hospital and the University of Miami Hospital. Women were asked to verbally consent prior to handing out surveys. Following completion of the surveys, women were given educational pamphlets from the CDC. Pregnant women above 17 years of age in all trimesters who could speak and read English were included. Minors, women that presented for a postnatal visit, and women who couldn't read or speak English were excluded. The surveys were entered into REDcap and analyzed using SAS Studio University Edition. Qualitative variables were described as percents for outcomes.

## Results

**Figure 1:**
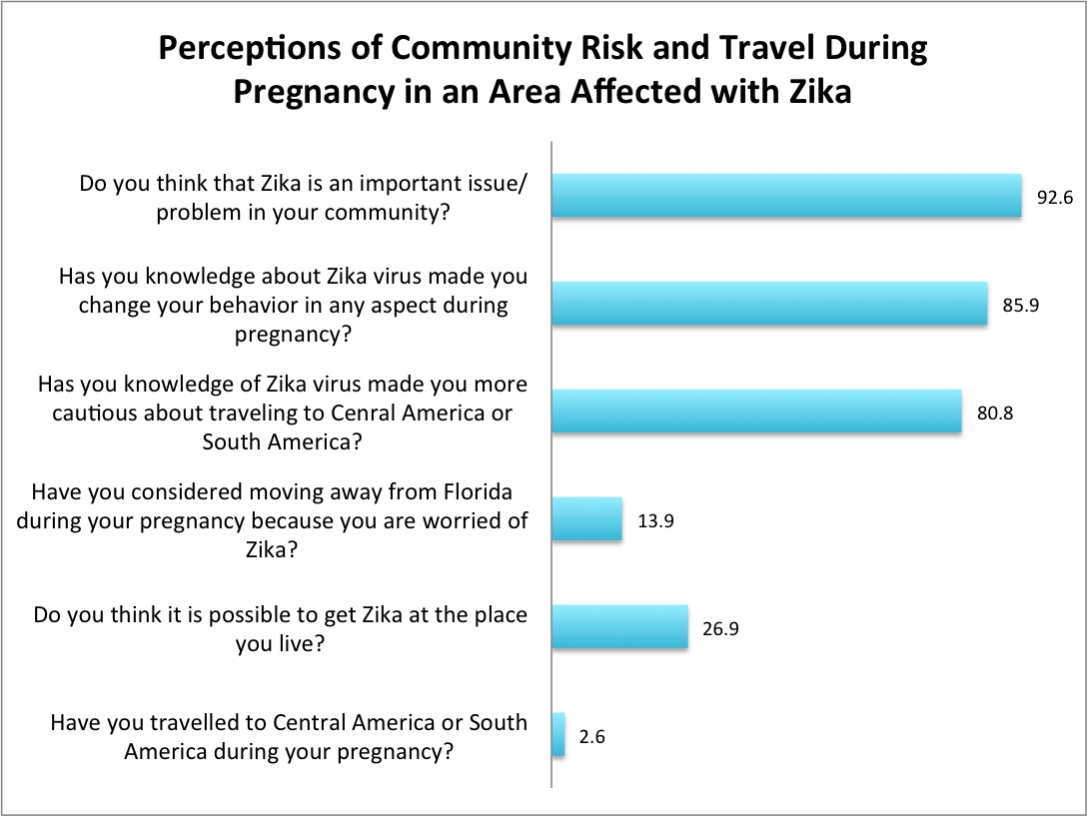
Perceptions of Community Risk and Travel During Pregnancy in an Area Affected with Zika

A total of 85 women were surveyed in the antenatal clinics at the University of Miami Hospital and the Jackson Memorial Hospital from January 27, 2017 to March 3, 2017. Overall, 92.6 percent of women believed Zika was an important issue in their community, while 85.9 percent reported changed behavior during pregnancy due to Zika. However, only 26.9 percent of women believed they could get Zika where they live, 13.9 percent considered moving away from Florida, 80.8 percent became more cautious about travel because of Zika, and only 2.6 percent traveled to other affected countries in Central America and South America during their pregnancy (Figure 1).

## Discussion

Between June 2016 and August 2016, the first cases of US-based transmissions of Zika were reported and Miami became an area of high risk for Zika [3]. Since then, the promotion of Zika awareness was done through advertising and social media [7]. In Florida, awareness and prevention was promoted through billboards, which advocated for the use of barrier protection or mosquito repellents [7]. The governor of Florida also called for protective measures against mosquitos, such as aerial spraying and higher research investments for treatment [8]. The combination of awareness efforts in Florida likely led to a very high number of women in Miami perceiving Zika to be an important issue in the community, resulting in increased cautiousness. In addition, a large number of women reported a change in behavior because of Zika, which was likely the result of the efforts made in advertising and social media.

Despite widespread awareness of Zika, only 26.9 percent of women believed they could get Zika in their community. This is a matter of concern because mosquitoes are prevalent year-round in Florida and tend to worsen during summertime [3]. In 2016, there were 218 cases of Zika that were locally acquired in Florida and 6 cases in Texas [3]. Whittmore et al. (N=99) found that 70.1 percent of women traveled to affected areas to visit their families despite being aware that they are at higher risk for Zika [5]. This may prove that although women are aware of Zika in affected areas, they are willing to overlook travel advisories to visit their families, which may be attributed to not completely understanding that they can indeed become infected with Zika in these locations. 

Till date, there are no studies evaluating the perceptions of the community risk of Zika in affected areas in the US. However, various studies have evaluated the perceptions of infection risk and travel to affected areas in pregnant women. Whittmore et al. found that 69.4 percent of responders were aware of government-issued travel advisories and 55.7 percent were aware that Zika affected the region [5]. Mouchtouri et al. performed a knowledge and attitudes survey of pregnant women (N=573) in Greece and found that women who traveled abroad in the past six months were more likely to answer questions about Zika transmission through mosquito bites correctly, but did not answer questions on the sexual transmission of Zika correctly. Interestingly, 14.4 percent of women believed it is safe for pregnant women to travel to affected regions and only 0.7 percent admitted they would visit a country affected by Zika [6].

## Conclusions

A majority of women in this study believed Zika is an important issue in their community. However, only a fourth of all women in this study believed they could contract Zika in their location. Efforts to educate pregnant women in affected areas about Zika prevention should be undertaken. Further studies comparing the perceptions of community risks and travel behaviors in other affected areas are warranted. 
